# SW-620 cells treated with topoisomerase I inhibitor SN-38: gene expression profiling

**DOI:** 10.1186/1479-5876-3-44

**Published:** 2005-12-23

**Authors:** Vinicius Souza, Yan Bin Dong, H Sam Zhou, Wolfgang Zacharias, Kelly M McMasters

**Affiliations:** 1 Division of Surgical Oncology, Department of Surgery, University of Louisville School of Medicine, Louisville, Kentucky, USA; 2 Department of Medical Oncology, University of Louisville School of Medicine, Louisville, Kentucky, USA; 3 James Graham Brown Cancer Center, Louisville, Kentucky, USA

## Abstract

**Background:**

The goal of this study was to evaluate changes in gene expression in SW-620 cells in response to SN-38 in order to further elucidate the mechanisms by which SN-38 causes apoptosis and cell cycle arrest.

**Methods:**

We used a quantitative gene expression microarray assay to identify the genes regulated by SN-38 treatment in colon cancer cells and confirmed our results with RT-PCR. By gene expression profiling, we first screened a proprietary list of about 22,000 genes.

**Results:**

Treatment with SN-38 cells resulted in two-fold or greater alteration in the level of expression of 192 genes compared to control treatment. Most of the affected genes were not known to be responsive to SN-38 prior to this study. SN-38 treatment of these cells was found to affect the expression of various genes involved in DNA replication, transcription, signal transduction, growth factors, cell cycle regulation, and apoptosis, as well as other genes with unknown function. Changes in expression of 14 genes were confirmed by quantitative real-time polymerase chain reaction (RT-PCR).

**Conclusion:**

This study leads to an increased understanding of the biochemical pathways involved in SN-38-induced apoptosis and possibly to the identification of new therapeutic targets.

## Introduction

Each year over 50,000 people in the United States die from colorectal cancer, which is the second leading cause of cancer death [1]. Although the mortality rate associated with colorectal cancer has been declining, in part due to early diagnosis and the introduction of new drugs, present therapy is often ineffective. Moreover, the majority of the colorectal cancer cases that are known to be hereditary do not show a clearly identifiable genetic etiology [2].

Irinotecan (CPT-11) is a chemotherapeutic prodrug that is converted to SN-38 (its most active metabolite) by carboxylesterases that are abundantly present in the liver [3]. SN-38 has high anti-tumor activity against refractory solid tumors, such as carcinomas of the lung, cervix, ovary, and colon [4]. SN-38 binds the transient cleavable complex between DNA and topoisomerase I, preventing dissociation of the DNA-topoisomerase I complex and thereby inducing cell cycle arrest and apoptosis. This mechanism of action of SN-38 was observed in colon cancer cell lines [5]. Topoisomerase I functions to unwind portions of the DNA during replication and transcription by making transient nicks followed by re-ligation in one strand of the DNA. SN-38 works to prevent topoisomerase I from resealing these DNA nicks. As replication continues, permanent single strand breaks in the DNA lead to cell death [6]. SN-38 effectiveness has been directly linked to high levels of topoisomerase I activity in the cell [7]. Since the activity of topoisomerase I is usually higher in the tumor cells than in normal cells, SN-38 action holds some degree of specificity to tumor cells [6]. Furthermore, a decrease in expression levels of topoisomerase I in colorectal cancer cell lines is believed to cause resistance against the effects of SN-38. In the present study, we investigated the changes in gene expression that accompany SN-38-induced colorectal cancer cell apoptosis to evaluate possible novel signaling pathways involved in this process.

## Materials and methods

### Cells and culture conditions

Human colon cancer cell line SW-620 was purchased from ATCC (Rockville, MD; ATCC number CCL-227). Cells were routinely propagated as a monolayer culture in Dubbelco's Modified Eagle Medium (Gibco, Invitrogen) supplemented with 10% heat-inactivated fetal bovine serum (FBS) and penicillin (100 U/ml)/streptomycin (100 μg/ml) solution. All cell culture reagents were obtained from Invitrogen/Life Technologies (Carlsbad, CA). SN-38 was purchased from Sigma (St. Louis, MO). Cells were cultured in a 5% CO_2 _incubator at 37°C and sub-cultured every 3 to 4 days (when about 80% confluent).

For experiments, SW-620 cells were plated for 48 h in 4 × 6-well plates (3 × 10^5 ^cells/ well). Cells were then exposed to SN-38 (50 nM concentration) for 18 h. The control sample was exposed to vehicle only. For further control of the effects of SN-38, SW-620 cells were also plated in 1 × 96-well plate (1 × 10^4 ^cells/well) for 48 h. Cells were then exposed to increasing concentrations of SN-38 (0, 12.5 nM, 25 nM, 50 nM, 100 nM, 200 nM) and samples were analyzed after 18 h, 92 h, 120 h, and 168 h.

### Microarray analysis

#### cRNA synthesis and labeling

At 18 h after drug exposure, total RNA was isolated with Trizol reagent (Gibco Life Technologies). We chose a time point prior to any evidence of apoptosis because we wanted to investigate the persistent changes in gene expression in response to SN-38 treatment. First- and second-strand cDNA were synthesized from 5–15 μg of total RNA using the SuperScript Double-Stranded cDNA Synthesis Kit (Gibco Life Technologies) and oligo-dT24-T7 (5'-GGC CAG TGA ATT GTA ATA CGA CTC ACT ATA GGG AGG CGG-3') primer, according to the manufacturer's instructions.

Next, cRNA was synthesized and labeled with biotinylated UTP and CTP by in vitro transcription using the T7 promoter coupled double-stranded cDNA as template and the T7 RNA Transcript Labeling Kit (ENZO Diagnostics Inc.). Briefly, ds cDNA synthesized from the previous steps were washed twice with 70% ethanol and resuspended in 22 μl of RNase-free H_2_O. The cDNA was incubated with 4 μl of 10× each reaction buffer, biotin-labeled ribonucleotides, DTT, RNAse inhibitor mix and 2 μl 20× T7 RNA polymerase for 5 h at 37°C. The labeled cRNA was separated from unincorporated ribonucleotides by passing through a CHROMA SPIN-100 column (Clontech) and precipitated at -20°C for 1 h to overnight.

#### Oligonucleotide array hybridization and analysis

The cRNA pellet was resuspended in 10 ul RNase-free H_2_O, and 10.0 μg was fragmented by heat and ion-mediated hydrolysis at 95°C for 35 min in 200 mM Tris-acetate, pH 8.1, 500 mM KOAc, and 150 mM MgOAc. The fragmented cRNA was hybridized for 16 h at 45°C to HG_U95Av2 oligonucleotide arrays (Affymetrix, Santa Clara, CA) containing 22,284 gene transcripts together with additional probe sets designed to represent EST sequences. Arrays were washed at 25°C with 6 × SSPE (0.9 M NaCl, 60 mM NaH_2_PO_4_, 6 mM EDTA + 0.01% Tween 20) followed by a stringent wash at 50°C with 100 mM MES, 0.1 M [Na+], and 0.01% Tween 20. The arrays were then stained with phycoerythrein-conjugated streptavidin (Molecular Probes), and the fluorescence intensities were determined using a laser confocal genearray scanner (Agilent). The scanned images were analyzed using Microarray Suite 5.0 or GCOS software (Affymetrix). Sample loading and variations in staining were standardized by scaling the average of the fluorescent intensities of all genes on an array to constant target intensity (250) for all arrays used. Data analysis was conducted using Microarray Suite 5.0 (Affymetrix) following user guidelines. The signal intensity for each gene was calculated as the average intensity difference, represented by [Σ (PM – MM)/(number of probe pairs)], where PM and MM denote perfect-match and mismatch probes. Several criteria were used to identify genes whose expression was affected by SN-38 treatment. First, whether the fold-change of each gene, as provided by the Affymetrics analysis, was more than 2-fold up- or down-regulation. The second criterion employed was whether the difference call, as provided by the GeneChip software was moderately induced (MI) or induced (I) for upregulated genes and moderately decreased (MD) or decreased (D) for downregulated genes. In addition, only genes in which absolute calls were double present (or one present – one absent in SN-38 versus control) were considered as SN-38 responsive genes. Two completely independent experiments were performed. Only genes that had changed in the same direction in both experiments with the change p value of < 0.006 were considered for further analysis. The final fold changes were calculated based on the average signal log ratio of the two experiments. The genes whose average expression differed by more than 2-, 5- or 10-fold when compared to the control treatment were grouped by functional category.

We used the on-line data mining tools from 3 different software programs: NetAffx Analysis Center (Affymetrix, Santa Clara, CA), GeneSpring (Silicon Genetics, Redwood City, CA), and Genesifter (VizX Labs, Seattle, WA).

#### RT-PCR

At 18 h after drug exposure, RNA was prepared using the RNeasy mini kit along with RNase-free DNase set to rid any traces of DNA contamination (both kits were from QIAGEN, Valencia, CA). cDNA was prepared from 500 ng of RNA (TaqMan Reverse Transcription Reagents, ABI/Roche, Branchburg, NJ), based on the manufacturer's instructions. The template cDNA (12.5 ng/sample) for control and SN-38 was mixed with Syber green master mix (ABI). Mixtures were aliquoted into 96-well optical reaction plates (ABI) along with forward and reverse primers for each target gene. Samples were run in duplicates. The ABI Prism 7000 sequence detection system was used. Primers were designed using ABI primer express software. Amplicon sizes were all below 200 bp. Data were analyzed using the comparative *C*_T _method, which the fold change of target gene samples (SN-38 treatment) normalized to the housekeeping gene (beta-actin) relative to the calibrator (control) is calculated by 2^-ΔΔCT ^equation, where ΔΔ*C*_T _= Δ*C*_T _(sample) - Δ*C*_T _(calibrator), and Δ*C*_T _is the *C*_T _value of the target gene subtracted from the *C*_T _value of the housekeeping gene, all determined in the exponential phase of the reactions [8]. (PCR primers are listed in Table 1).

#### Trypan blue cell count and cell cycle analysis

At 18 h after drug exposure, cells from two wells of each treatment were harvested, and half of them were stained with trypan blue solution 0.4% (Sigma Chemical Co.) for 5 min. Cells from each of the samples then were counted using a hemacytometer. The other half of each of the two samples was washed once with PBS and fixed in 70% (vol/vol) ethanol at -20°C for up to one week. Cells were pelleted, washed once with PBS, and resuspended in propidium iodide (PI) solution (50 μg/ml PI, 0.5 mg/ml RNase in PBS, pH 7.4) for 30 min in the dark. Flow cytometry analysis was performed at 18 h after treatment. FACS analysis was performed on a FACScan flow cytometer (Becton Dickinson, San Jose, CA, USA). The data from 10,000 cells were collected and analyzed using CellFIT cell-cycle analysis software (version 2.01.2).

#### MTT assay

The MTT assay is also used for the measurement of cytotoxicity of a drug. Briefly, SW-620 cells were plated into 96-well plates and treated with increasing concentration of SN-38 (0 nM, 12.5 nM, 25 nM, 50 nM, 100 nM, 200 nM). We used 1e4 cells in 100 μl of DMEM 10%FBS per well. After 18 h, 24 h, 96 h, and 120 h of incubation at 37°C, 10 μl MTT (5 mg/ml, Sigma, St. Louis, MO) was added to each well and incubated at 37°C for 4 h. After incubation, 50 μl lysing buffer (10%SDS in 0.01 N HCl) was added, and samples were incubated at 37°C overnight for complete cell lysis. On the following day, cytotoxicity was assessed by measuring the conversion of the tetrazolium salt MTT to formazan through measurement of absorbance at 570 nm.

## Results

### Exposure to SN-38 alters the gene expression in SW-620 colon cancer cells

At 18 h after treatment, flow cytometry analysis demonstrated no significant change in G2 cell cycle arrest between treatment and control. At the same time point, MTT analysis did not demonstrate any significant difference in cell viability. After 4 days, MTT analysis showed a 30% decrease in cell proliferation in treated cells compared to the control. Our results are consistent with past studies that showed SN-38 induces apoptosis in cells with dysfunctional *p53 *gene (SW-620 cells are *p53 *mutant) [6,9].

In our microarray analysis, after 18 h of SN-38 exposure, 192 genes demonstrated over a 2-fold change in mRNA expression. Forty of these 192 genes were downregulated, whereas 152 genes were upregulated. Among those genes, we found 13 genes with expression change greater than 5-fold: 10 upregulated and 3 downregulated. Next, we used NetAffx analysis to categorize all of the altered genes based on their biological processes and molecular functions, and we found most of the genes to be related to transcription, DNA replication, signal transduction, growth factors, cell cycle regulation, and apoptosis. All but one of the genes related to apoptosis and DNA replication were upregulated (*FAF1 *was downregulated).

**Figure 1:**
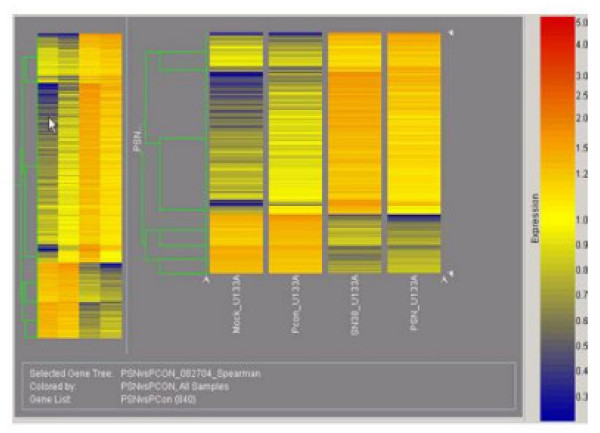
***The two independent microarray experiments behaved similarly: ***Genes demonstrated in this gene tree contain 840 genes that changed with a p value < 0.006 in either experiment, indicating whether the genes in the two experiments were upregulated or downregulated in the same direction in both experiments. GeneSpring software (version 6.1) was used for this gene tree (Silicon Genetics, Redwood City, CA). Values below 0.01 were set to 0.01. Each measurement was divided by the 50th percentile of all measurements in that sample. Each measurement for each gene in the specific samples was divided by the median of that gene's measurements in the corresponding control samples. (A full description of these genes and detail of analysis is available on the GEO repository web site, attachment to Acc. #GSM24425-28). Mock and Pcon are the control sample for experiment 1 and experiment 2, respectively. SN-38 and PSN are the treatment sample for experiment 1 and 2, respectively.

Little variation was observed in the 840 genes that changed in both microarray experiments (Fig. 1). We found 192 genes that had altered expression over 2-fold and changed in the same direction and with similar fold changes. In addition, genes that had more than one set of probes represented in the microarray chip had the same expression pattern. We used Miroarray Suite 5 (MAS 5), Data Mining Tool (DMT), and NetAffx Analysis Center to categorize the genes according to their biological and molecular functions. The vast majority of the genes fell into four categories: cell cycle, apoptosis, signal transduction, and transcription.

**Table 1:** *Verification of microarray analysis by Real Time – Polymerase Chain Reaction*: In order to evaluate the SN-38-mediated activation of pro-apoptotic pathways, we confirmed the microarray results with real-time PCR using total RNA from 18 h post SN-38 exposure of the SW-620 cells and control. Primer express software from ABI has been used to design the primers for real-time PCR (amplicon sizes were about 70 bp). Gene symbols and Probe Set ID numbers are as found at the NCBI website  and NetAffx™ Analysis Center . Data are averages from two separate experiments, each in duplicate. Data are reported as the fold change of gene expression level SN-38 treated cells versus no treatment control. FC = fold change.

Gene Symbol	Affymetrix Probe Set ID	Primer for RT-PCR	RT-PCR avg. FC	Microarray avg. FC
β-actin	NM_001101	F: CGATCCACACGGAGTACTTG R: GGATGCAGAAGGAGATCACTG	NC	NC
*RGC32*	218723_s_at	F: GAAGCCTTCATTGCTGATCTTG R: GTCCTCGGAACTTTCTGAAGCT	6.2	6.5
ITGB7	205718_at	F: CGGCTCTCGGTGGAAATCTAT R: GGTCGTGATGGCACTTTTGTAG	3.1	7.8
PDCD4	202731_at	F: GTGCTGGAGCGGTTTGTAGAA R: CCTTCGCTTACAAAACGCTTTC	1.8	2.5
ABAT	209459_s_at	F: CTGTTGGTTCAACGTGGTTTCC R: GCATTTCGGTATCATCTGCCTG	3.4	6.7
DKK3	214247_s_at	F: ACCCCTGTCCAGATTATTGGC R: TTGTTTCCATCTCCTCCCCTC	4.4	6
LYZ	213975_s_at	F: CGCTACTGGTGTAATGATGGCA R: CAAGCTACAGCATCAGCGATGT	4.6	11
CCL20	205476_at	F: GTCTGTGTGCGCAAATCCAA R: CCATTCCAGAAAAGCCACAGTT	3.6	10.6
UBD	205890_s_at	F: TTGCAATGGAAAGAGACTGGAA R: AGGAAGAGTAAGTTGCCCTTTCTG	28.2	17.9
FGF3	214571_at	F: AATCAGGGTCCAGTGGGAACT R: CAAATGCCCTGCATTGCA	-12.6	-5.1
FAF1	218080_x_at	F: CGGTGGAACCAGCCATTC R: TTGGCGAGGAGCCCTTCT	-4.8	-5.1
HIST1H2BK	208527_x_at	F:TACGTGTACAAGGTGCTGAAACAG R: CATGATCCCCATGGCTTTAGA	-8.6	-6.1
Cyclin E1	213523_at	F: CACCAGCCACCTCCAGACA R: GTGGGAGTCCCTTAGGTCAACTAG	3.1	2.2
Cyclin E2	205034_at	F:GAAGTAGCCGTTTACAAGCTAAGCA R: GCCTGGATTATCTGGGCTTCT	3.2	4.1
TNFRSF6	216252_x_at	F: AATCATCAAGGAATGCACACTCA R: AAGCCACCCCAAGTTAGATCTG	4.2	4.5

### Verification of microarray analysis by quantitative RT-PCR

RT-PCR was performed to confirm the changes in gene expression observed in the microarray analysis. RNA samples from all three treatments in each of the two experiments were used to confirm changes. We selected genes that either had a high fold change in both microarray experiments or were directly related to apoptotic pathways and cell cycle regulation. For all 14 genes tested, results from microarray and RT-PCR were in agreement (primers are listed in Table 1). Some genes that had a high fold change in only one microarray experiment were also tested. For each of these genes, no change was observed on RT-PCR.

### Genes involved in apoptotic pathways

Several genes directly involved in apoptotic pathways were significantly altered. Different isoforms of the *TNFRSF6 *gene [tumor necrosis factor (TNF) receptor] were found to be upregulated. This result is in agreement with suggestions that SN-38 increases sensitivity of the cells to TNF-related apoptosis-inducing ligand (TRAIL) [10]. Other studies detected upregulation of several genes in the TNF family as early as 2 h after exposure to SN-38 [11]. This indicates that changes in the expression of TNF-related genes are persistent, and these changes may play an essential role in induction of apoptosis. Intriguingly, we found that Fas associated factor-1 (*FAF-1*), a Fas-binding pro-apoptotic protein, was downregulated, suggesting that it does not play a direct role in apoptosis induced by SN-38 [12-14]. It has been shown that *Bax *activation, a pro-apoptotic member of the *Bcl-2 *family, is required for TRAIL-mediated apoptosis in colon cancer cells (HCT116) [15-17]. Other studies have also demonstrated *Bax *upregulation after SN-38 treatment in colorectal cancer cells [18]. In conformity to these studies, we found that *Bax *expression was 2.5-fold upregulated in our experiment. In addition, *BNIP3L*, a homolog of the pro-apoptotic protein BNIP3, was slightly upregulated. Overexpression of both *BNIP3L *and *Bax *suggest that SN-38 may act via the *Bax*-related apoptotic pathway.

## Discussion

Our results showed an increase in expression of three genes in the cyclin family: *Cyclin E1 (CCNE1), Cyclin E2 (CCNE2)*, and *Cyclin G2 (CCNG2)*. Cyclins are involved in cell cycle regulation and tumor growth. Previously, Ueno et al. [19] demonstrated *Cyclin E *expression is enhanced after colorectal cancer cells are exposed to SN-38 and suggested that upregulation of *Cyclin E *expression might trigger induction of apoptosis. Our results also showed a mild upregulation in a variety of cyclin-dependent kinase (Cdk)-related genes, and significant overexpression of *RGC32*, that encodes a protein involved in the regulation of cyclin-dependent kinase activity that is known to increase DNA synthesis in glioma cells [20]. It has been shown that flavopiridol, a cyclin-dependent kinase inhibitor, when used in combination with SN-38, synergistically increases cell death in colon cancer cells [9]. Our findings suggest flavopiridol might enhance the apoptotic effects of SN-38 in tumor cells by inhibiting Cdk and Cdk-related gene activity. Further experiments are necessary to evaluate the role of Cdk in resistance to SN-38 therapy and its importance in the regulation of colon cancer cell death.

SN-38 reduces tumor growth by inhibiting topoisomerase I function. Interestingly, studies have shown no change in the level of topoisomerase I mRNA following SN-38 treatment [9,21]. Our results were consistent with these studies showing that topoisomerase I mRNA upregulation by SN-38 is not necessary to achieve drug-induced apoptosis. However, we identified several other genes, not recognized in previous studies, which may be SN-38 target candidates. We identified 13 genes with expression altered by more than 5-fold. The gene with the highest upregulation is *UBD *(*ubiquitin D*, 17.9-fold). Although further studies are needed to investigate the role of *UBD *in inhibiting cell growth in SN-38-treated SW620 cells, *UBD *expression may increase via the proteasome pathway degradation of proteins that induce cell growth. The second highest expressed gene in SN-38 cells compared to controls is *LYZ *(lysozyme involved in carbohydrate metabolism) with an 11-fold change, and third is *CCL20 *(a chemokine ligand) with 10.6-fold change. Previous studies have demonstrated that overexpression of chemokines (including *CCL-20*) in murine tumor cells can induce anti-tumor T-cell responses by recruiting immature dendritic cells [22,23]. Further studies are necessary to determine whether *CCL-20 *behaves in a similar fashion in human cancer cells. Other genes significantly upregulated were *ITGB7 *(involved in integrin-mediated cell adhesion), *ABAT *(involved in metabolism, regulation of transcription and GENMAPP/KEGG pathway), *DKK3 *(a regulator of the Wnt receptor signaling pathway involved in development and morphogenesis), *PROCR *(encodes a receptor for activated protein C), *SGK *(involved in protein aa phosphorylation, sodium ion transport, response to stress, and apoptosis), and *SMARCA1 *(which is thought to regulate the transcription of other genes by interfering with chromatin structure). Lastly, *HIST1H2BE*, which encodes a component of histones, was downregulated 6.1-fold.

## Conclusion

Our data confirm and extend the findings of other investigators and introduces several possible novel pathways to explain the mechanisms used by SN-38 to induce cell arrest and apoptosis. SN-38-induced upregulation of cell signaling receptors may increase the tumor cells' sensitivity to pro-apoptotic factors, such as TRAIL. Further studies will be necessary to elucidate the pathways by which SN-38 signals cells to undergo either cell arrest or apoptosis. Elucidation of these pathways may lead to the development of new drugs to overcome tumor cell resistance to SN-38 treatment.

## Abbreviations

CPT-11, irinotecan; RT-PCR, real-time polymerase chain reaction; FBS, fetal bovine serum; PI, propidium iodide.

## Competing interests

The author(s) declare that they have no competing interests.

## Authors' contributions

VS carried out the gene expression profiling and drafted and submitted the manuscript. YD and HSZ participated in the design of the study. WZ participated in the microarray experiments. KMM conceived of the study and participated in its design and coordination. All authors read and approved the final manuscript.
